# Blood urea nitrogen-to-serum albumin ratio as a predictor of long-term mortality in patients with acute heart failure

**DOI:** 10.3389/fcvm.2026.1769180

**Published:** 2026-03-25

**Authors:** Lixin Jia, Yangkai Fan, Bokang Qiao, Haichu Wen, Jie Du

**Affiliations:** 1 Department of Cardiology, Beijing Anzhen Hospital, Capital Medical University, Beijing, China; 2 Beijing Anzhen Hospital, Capital Medical University, Beijing, China; 3 The Key Laboratory of Remodeling-Related Cardiovascular Diseases, Ministry of Education, Beijing Collaborative Innovation Center for Cardiovascular Disorders, Beijing Institute of Heart, Lung & Blood Vessel Disease, Beijing, China

**Keywords:** acute heart failure, blood urea nitrogen to serum albumin ratio, cohort study, mortality, risk stratification

## Abstract

**Objectives:**

The objective of this study was to evaluate the baseline blood urea nitrogen-to-serum albumin ratio (BAR) in patients with acute heart failure (AHF) upon admission as a predictor of long-term mortality.

**Methods:**

In this retrospective cohort study, a total of 2,556 patients who were hospitalized for AHF at Beijing Anzhen Hospital between 2017 October and 2020 January were enrolled. Multivariate Cox regression analysis was performed to explore the association between BAR and out-of-hospital all-cause mortality. The risk stratification capability of BAR was also calculated using Kaplan–Meier survival analysis.

**Results:**

Of the 2,556 patients with AHF, 1,196 had with heart failure with reduced ejection fraction, 1,066 had heart failure with preserved ejection fraction, and 294 had heart failure with mildly reduced ejection fraction. After a median follow-up of 1.84 years (IQR: 1.15, 2.57), 665 (26.0%) patients experienced out-of-hospital all-cause mortality. Univariate Cox regression analysis revealed that BAR was significantly associated with long-term mortality in all patients with AHF (*p* < 0.05). After adjusting for age, sex, and traditional clinical risk factors, BAR remained independently associated with mortality in both the overall population and each heart failure subgroup.

**Conclusion:**

BAR was associated with long-term mortality in patients with AHF after discharge, regardless of left ventricular ejection fraction at admission. BAR exhibited risk stratification capacity for long-term mortality outcomes in patients with AHF.

## Introduction

Heart failure (HF) affects approximately 1%–2% of the global population, with prevalence increasing to 10% among people over 70 years of age (1). Acute heart failure (AHF), a serious medical condition, is defined as the new onset or worsening of symptoms and signs of HF (2). As a common cause of hospital admission, AHF is associated with high mortality and hospital readmission rates (3). It is estimated that in-hospital mortality for AHF is around 4%, rising to 25%–30% at 1 year (4, 5). Over the past decade, advances in the treatment of HF—including heart transplantation and left ventricular assist device (LVAD) implantation—have greatly improved clinical outcomes for patients (6). Therefore, identifying high-risk patients with AHF and poor prognosis is crucial in clinical practice.

Several studies have identified the predictors of 180-day mortality in AHF, including malignancy, severe lung disease, smoking history, systolic blood pressure, and heart rate (7). In addition to these factors, biomarkers have become valuable tools for AHF prognosis (8). Established prognostic biomarkers for AHF include natriuretic peptides, cardiac troponin, and soluble suppressor of tumorigenicity-2 (sST2), each with different abilities and limitations. However, given the complex pathophysiology of AHF, no single biomarker is appropriate for all patients with AHF. Therefore, identifying novel biomarkers for short- and long-term prognoses in AHF is essential to guide treatment strategies and improve patient outcomes.

The blood urea nitrogen-to-serum albumin ratio (BAR) has been recognized as an important prognostic biomarker in several medical conditions, including sepsis (9), lung cancer (10), and myocardial infarction (11). As a simple and convenient biomarker, BAR reflects renal function, nutritional status, liver function, and systemic inflammation, all of which are key prognostic factors in HF. A retrospective study demonstrated that BAR is an independent risk factor for in-hospital and 90-day mortality in critically ill patients with chronic HF admitted to the intensive care unit (12). However, its long-term predictive value remains unknown.

In the present study, we evaluated the predictive value of BAR for long-term mortality in patients with AHF, aiming to provide novel insights into its potential role as a practical biomarker.

## Methods

### Study population

The study is part of the Registry Study of Biomarkers in HF (BIOMS-HF), an observational, retrospective study. From May 2017 to July 2021, a total of 2,556 patients with AHF admitted to Beijing Anzhen Hospital were enrolled. Among them, 1,196 patients were diagnosed with heart failure with reduced ejection fraction (HFrEF), 1,066 with heart failure with preserved ejection fraction (HFpEF), and 294 with heart failure with mildly reduced ejection fraction (HFmrEF).

The inclusion criteria were as follows: (1) age ≥18 years; (2) BNP or N-terminal pro-BNP concentrations >35 or 125 pg mL^−1^, respectively; and (3) HF symptoms and signs (e.g., dyspnea at rest or with minimal exertion, dry–wet rales, hydrothorax, ascites, peripheral edema, and pulmonary congestion on X-ray radiography).

The exclusion criteria were as follows: (1) end-stage renal disease requiring dialysis; (2) active malignancy or life expectancy <6 months; and (3) inability to provide informed consent.

The study was conducted in accordance with the principles of the Declaration of Helsinki and was approved by the Beijing Anzhen Hospital Ethics Committee. Informed consent was obtained from all participants. More details are available at ClinicalTrials.gov (NCT03784833).

### Blood sample and clinical data collection

Blood samples were collected in the morning following a 12- to 16-h overnight fast and drawn into coagulation-promoting tubes. Samples were centrifuged at 3,000 rpm for 10 min in the clinical laboratory. The supernatant serum was quickly removed, aliquoted, and stored at −80°. All clinical data were extracted and identified from electronic health records, including clinical symptoms, demographics, comorbidities, blood pressures, heart rate, echocardiographic measurements, and laboratory results. BAR was calculated from the ratio of BUN (mg/dL) to albumin (g/dL).

### Definition of outcome and follow-up

Clinical outcomes were tracked from the date of enrollment until the occurrence of the clinical endpoint, all-cause mortality, or the end of the 4-year study period, whichever came first. Follow-up information was obtained through telephone interviews and clinic visits. Outcomes were determined by two cardiologists. If there was disagreement about the final diagnosis, a third cardiologist served as arbitrator.

### Statistical analyses

Continuous variables were presented as median and interquartile range, and compared using the Mann–Whitney *U*-test. Categorical variables were presented as counts with percentages, and compared using the chi-square test or Fisher's exact test.

Cox proportional hazards regression was used to evaluate the associations between risk factors and out-of-hospital all-cause mortality, with hazard ratios (HRs) calculated after verifying the proportional hazards assumption. In the crude model, we evaluated the association between serum ln BAR levels and primary endpoints. Multivariable model 1 was adjusted for sex and age, while multivariable model 2 was adjusted for sex, age, smoking, diabetes mellitus, prior myocardial infarction, prior revascularization, BNP, high-density lipoprotein (HDL) cholesterol, low-density lipoprotein (LDL) cholesterol, creatinine (Cr), use of angiotensin-converting enzyme inhibitor (ACEI) or angiotensin II receptor blocker (ARB), use of beta-blockers, and use of diuretics. Multicollinearity among all covariates was formally assessed using variance inflation factors (VIFs), with a VIF value <5 considered indicative of the absence of significant structural multicollinearity. Spearman correlation analyses were performed to estimate the correlation coefficients among BAR and clinical risk factors.

To evaluate the incremental prognostic value of BAR relative to its individual components, we performed head-to-head model comparisons using a substitution approach. In particular, a BUN-based model and an albumin-based model—both adjusted for the same set of baseline covariates—served as references to evaluate the discriminative and reclassification performance of the BAR-based model. Discriminative ability was compared using the DeLong test for C-indices, while improvement in risk reclassification was quantified using continuous net reclassification improvement (NRI) and integrated discrimination improvement (IDI).

Survival probability was estimated using Kaplan–Meier survival curves according to BAR tertiles, and the differences between the three groups were evaluated using the log-rank test.

Sensitivity analyses were conducted to verify the robustness of our findings. To clarify whether BAR provides independent prognostic information beyond established cardiorenal biomarkers, we constructed separate multivariable models by systematically excluding serum creatinine or BNP from the adjustment framework to observe the stability of the hazard ratios for BAR.

## Results

### Baseline characteristics

A total of 2,556 patients with HF were enrolled, comprising 1,196 with HFrEF, 1,066 with HFpEF, and 294 with HFmrEF. During a median follow-up period of 1.84 years (IQR: 1.15, 2.57), 665 patients (26.0%) experienced out-of-hospital all-cause mortality.

The baseline characteristics of the study population are provided in Table 1. Compared with the survivors, non-survivors were significantly older and had a higher proportion of NYHA class III–IV symptoms at admission. Moreover, these patients exhibited greater comorbidities, including diabetes mellitus and chronic kidney disease. Notably, the non-survivors demonstrated significantly elevated levels of BNP and BUN, along with lower left ventricular ejection fraction (LVEF) and serum albumin concentrations.

**Table 1:** Baseline characteristics of patients with HF.

Clinical parameters	Non-death group (*n* = 1,891)	Death group (*n* = 665)	*p*-Value
Clinical characteristics			
Sex (male)	1,201 (63.5%)	415 (62.4%)	0.644
Age (years)	63.1 (14.7)	70.0 (13.1)	<0.001
NYHA class (*n*, %)			<0.001
II	463 (24.5)	99 (14.9)	
III–IV	1,428 (75.5)	566 (85.1)	
Smoking (*n*, %)	459 (24.3)	122 (18.3)	0.002
SBP (mmHg)	127 (24.2)	121 (24.3)	<0.001
DBP (mmHg)	75.5 (16.0)	70.7 (15.2)	<0.001
Medical history (*n*, %)			
Peripheral artery disease	56 (2.96)	28 (4.21)	0.153
Diabetes mellitus	635 (33.6)	261 (39.2)	0.01
Hypertension	1,092 (57.7)	396 (59.5)	0.445
Hyperlipidemia	680 (36.0)	206 (31.0)	0.023
Chronic kidney disease	325 (17.2)	208 (31.3)	<0.001
Atrial fibrillation	574 (30.4)	213 (32.0)	0.449
Prior myocardial infarction	366 (19.4)	147 (22.1)	0.142
Prior stroke	243 (12.9)	115 (17.3)	0.006
Prior revascularization	326 (17.2)	102 (15.3)	0.285
Echocardiogram			
LVEF (%)	44.7 (15.2)	43.0 (15.6)	0.016
E peak (cm/s)	106 (49.1)	110 (60.9)	0.143
A peak (cm/s)	83.9 (36.8)	86.5 (37.2)	0.122
E/A	1.51 (1.05)	1.50 (1.00)	0.875
LVEDD (mm)	56.1 (10.8)	55.0 (11.6)	0.039
LVESD (mm)	42.6 (12.5)	41.6 (13.0)	0.102
Laboratory examination			
BNP (pg/mL)	589 (289; 1,226)	1,033 (494; 2,089)	<0.001
HDL cholesterol (mg/dL)	1.04 (0.45)	0.97 (0.32)	<0.001
LDL cholesterol (mg/dL)	2.49 (0.89)	2.36 (0.87)	0.001
hsCRP (mg/L)	8.82 (11.1)	11.3 (12.0)	<0.001
Na (mmol/L)	139 (4.08)	138 (5.09)	<0.001
K (mmol/L)	4.21 (0.57)	4.37 (0.76)	<0.001
Cr (μmoI/L)	83.1 (66.9; 110)	101 (73.2; 153)	<0.001
UA (μmoI/L)	418 (332; 526)	456 (360; 570)	<0.001
Glucose (mmol/L)	6.76 (5.57; 8.96)	7.60 (5.93; 10.6)	<0.001
Hb (g/L)	134 (23.9)	121 (25.5)	<0.001
ALB (g/dL)	38.8 (6.92)	36.1 (7.13)	<0.001
UREA (mg/dL)	8.95 (7.56)	12.4 (8.19)	<0.001
BUN (mg/dL)	2.57 (2.00; 3.46)	3.50 (2.50; 5.50)	<0.001
BAR	0.07 (0.05; 0.10)	0.11 (0.07; 0.18)	<0.001
Cardiac medication (*n,* %)			
Use of statin	926 (49.0)	304 (45.7)	0.162
Use of ACEI or ARB	525 (27.8)	152 (22.9)	0.016
Use of beta-blockers	1,126 (59.5)	344 (51.7)	0.001
Use of spironolactone	878 (46.4)	290 (43.6)	0.226
Use of diuretic	1,220 (64.5)	446 (67.1)	0.254
Use of digxin	425 (22.5)	139 (20.9)	0.431
Use of ARNI	354 (18.7)	108 (16.2)	0.170

SBP, systolic blood pressure; DBP, diastolic blood pressure; NYHA, New York Heart Association; LVEF, left ventricular ejection fraction; LVEDD, left ventricular end-diastolic diameter; LVESD, left ventricular end-systolic diameter; BNP, brain natriuretic peptide; HDL, high-density lipoprotein; LDL, low-density lipoprotein; hsCRP, high-sensitivity C-reactive protein; Cr, creatinine; UA, uric acid; Hb, hemoglobin; ALB, albumin; ACEI, angiotensin-converting enzyme inhibitor; ARB, angiotensin II receptor blocker; ARNI, angiotensin receptor-neprilysin inhibitor.

*p*-Values were obtained through Mann–Whitney *U*-test for continuous variables and chi-square test or Fisher's exact test for categorical variables.

### Association of BAR with out-of-hospital all-cause mortality in AHF

To evaluate the prognostic value of BAR in patients with AHF, we assessed its association with mortality in the BIOMS-HF cohort. Univariate Cox regression analysis demonstrated that BAR was significantly associated with mortality in the overall HF population [HR (95% confidence interval (CI) = 1.94 (1.76–2.13)), *p* < 0.001]. This association remained significant across all HF subtypes, including HFrEF, HFpEF, and HFmrEF (Table 2).

**Table 2:** Multivariate-adjusted hazard ratios for primary endpoint associated with plasma concentrations of ln BAR (*n* = 2,556).

Number of events (%)		Crude model		Multivariable 1^a^		Multivariable 2^b^	
		HR^c^ (95% CI)	*p*–Value	HR^c^ (95% CI)	*p*-Value	HR^c^ (95% CI)	*p-*Value
All	665 (26.0%)	1.94 (1.76–2.13)	<0.001	1.88 (1.70–2.07)	<0.001	1.61 (1.43–1.61)	<0.001
HFrEF	322 (26.9%)	1.98 (1.70–2.30)	<0.001	1.87 (1.60–2.19)	<0.001	1.33 (1.09–2.05)	0.004
HFmrEF	81 (27.6%)	1.99 (1.53–2.60)	<0.001	1.91 (1.44–2.53)	<0.001	1.63 (1.16–2.28)	0.004
HFpEF	262 (24.6%)	1.90 (1.65–2.19)	<0.001	1.87 (1.61–2.17)	<0.001	1.83 (1.54–2.19)	<0.001

ACEI, angiotensin-converting enzyme inhibitor; ARB, angiotensin II receptor blocker; ARNI, angiotensin receptor-neprilysin inhibitor; HFrEF, heart failure with reduced ejection fraction; HFmrEF, heart failure with mildly reduced ejection fraction; HFpEF, heart failure with preserved ejection fraction; LDL, low-density lipoprotein.

^a^ Multivariable 1 was adjusted for sex and age.

^b^ Multivariable 2 was adjusted for sex, age, smoking, diabetes mellitus, prior myocardial infarction, prior revascularization, BNP, HDL cholesterol, LDL cholesterol, Cr, use of ACEI or ARB, use of beta-blockers and use of diuretic.

^c^ Hazard ratios (HRs) and 95% confidence intervals (CIs) were estimated by plasma ln BAR levels.

In multivariable model 1, after adjusting for age and sex, BAR maintained a significant association with mortality in both the overall cohort and all three HF subtypes. Further adjustment for previously reported risk factors of adverse outcomes and baseline covariates with significant differences (multivariable model 2) confirmed that BAR remained independently associated with mortality in the overall population and each HF subgroup (Table 2).

To ensure the stability of our multivariable Cox regression models, we performed formal multicollinearity diagnostics using VIFs. All covariates in model 2—including BAR, serum creatinine, and NT-proBNP—exhibited VIF values well below the threshold of 5, indicating the absence of significant structural multicollinearity (Table 3).

**Table 3:** Multicollinearity diagnostics for the clinical covariates.

Covariates	VIF	Tolerance (1/VIF)
BAR	1.38	0.73
BUN	1.45	0.69
Cr	1.23	0.81
Prior MI	1.20	0.83
Sex	1.18	0.85
Smoking	1.17	0.86
Prior revascularization	1.16	0.86
BNP	1.16	0.86
Age	1.14	0.88
*β* Blocker	1.10	0.91
Diabetes mellitus	1.09	0.92
Use of ACEI or ARB	1.07	0.93
LDL	1.06	0.94
Use of diuretics	1.04	0.96
HDL	1.04	0.96

BNP, brain natriuretic peptide; HDL, high-density lipoprotein; LDL, low-density lipoprotein; Cr, creatinine; MI, myocardial infarction; ACEI, angiotensin-converting enzyme inhibitor; ARB, angiotensin II receptor blocker; ARNI, angiotensin receptor-neprilysin inhibitor.

### Correlation among BAR and clinical risk factors

To elucidate the potential mechanisms underlying the association between BAR and mortality in patients with HF, we examined correlations with established indicators of left ventricular function/structure and traditional clinical risk factors. The results revealed that BAR was associated with age, sex, BNP levels, and LVEF (all *p*-values <0.05; Table 4). These findings suggest that BAR may reflect underlying abnormalities in left ventricular function and structure, thereby contributing to its association with adverse clinical outcomes in patients with HF.

**Table 4:** Correlation among BAR and clinical risk factors.

Clinical variables	Correlation coefficient (*r*)	*p*-Value
Age	0.16	<0.0001
Sex	0.06	0.0028
BNP	0.33	<0.0001
LVEF	−0.07	0.0009
LVEDD	0.01	0.4657

BNP, brain natriuretic peptide; LVEF, left ventricular ejection fraction; LVEDD, left ventricular end-diastolic diameter.

Spearman correlation analyses were used to estimate the correlation coefficients among BAR and clinical risk factors.

### Incremental prognostic value of BAR over traditional risk factors

To further evaluate whether BAR provides incremental prognostic value beyond its individual components, we constructed three parallel multivariable Cox models. These models were adjusted for established risk factors and baseline covariates that showed significant differences in our univariate analysis. In these analyses, a BUN-based model and an albumin-based model served as references to evaluate the performance of the BAR-based model. As shown in Table 5, the BAR-based model demonstrated a significant improvement in risk discrimination and reclassification compared to the BUN-based model [ΔC-index: 0.013 (0.005–0.021), *p* < 0.05; Continuous NRI: 0.242 (0.145–0.338)]. Notably, while the increase in C-index for the BAR-based model did not reach statistical significance when compared with the albumin-based model [ΔC-index: 0.004 (−0.001 to 0.009), *p* = 0.14], the BAR-based model yielded a significant improvement in risk reclassification, as evidenced by a positive continuous NRI of 0.138 (95% CI: 0.063–0.213). These findings indicate that although the discriminative power (C-index) of BAR and albumin is comparable, the integration of BUN into the ratio (BAR) allows for more precise risk stratification and net reclassification of patients across the risk spectrum.

**Table 5:** Comparison of BAR versus individual components in prognostic performance.

Comparison (reference model vs. new model)	ΔC-index (95% CI)^a^	*p*-Value	Continuous NRI (95% CI)	IDI (95% CI)
BUN vs. BAR	0.013 (0.005–0.021)	0.02	0.242 (0.145–0.338)	0.001 (0.000–0.002)
Albumin vs. BAR	0.004 (−0.001 to 0.009)	0.14	0.138 (0.063–0.213)	0.000 (0.000–0.001)

BAR, BUN-to-albumin ratio; CI, confidence interval; IDI, integrated discrimination improvement; NRI, net reclassification improvement.

All models were adjusted for age, sex, BMI, smoking, hypertension, diabetes, prior MI, LVEF, NT-proBNP, and creatinine.

^a^ ΔC-index represents the absolute increase in C-index when using BAR instead of BUN or albumin in the same multivariable framework.

### Risk stratification capability of BAR

**Figure 1:**
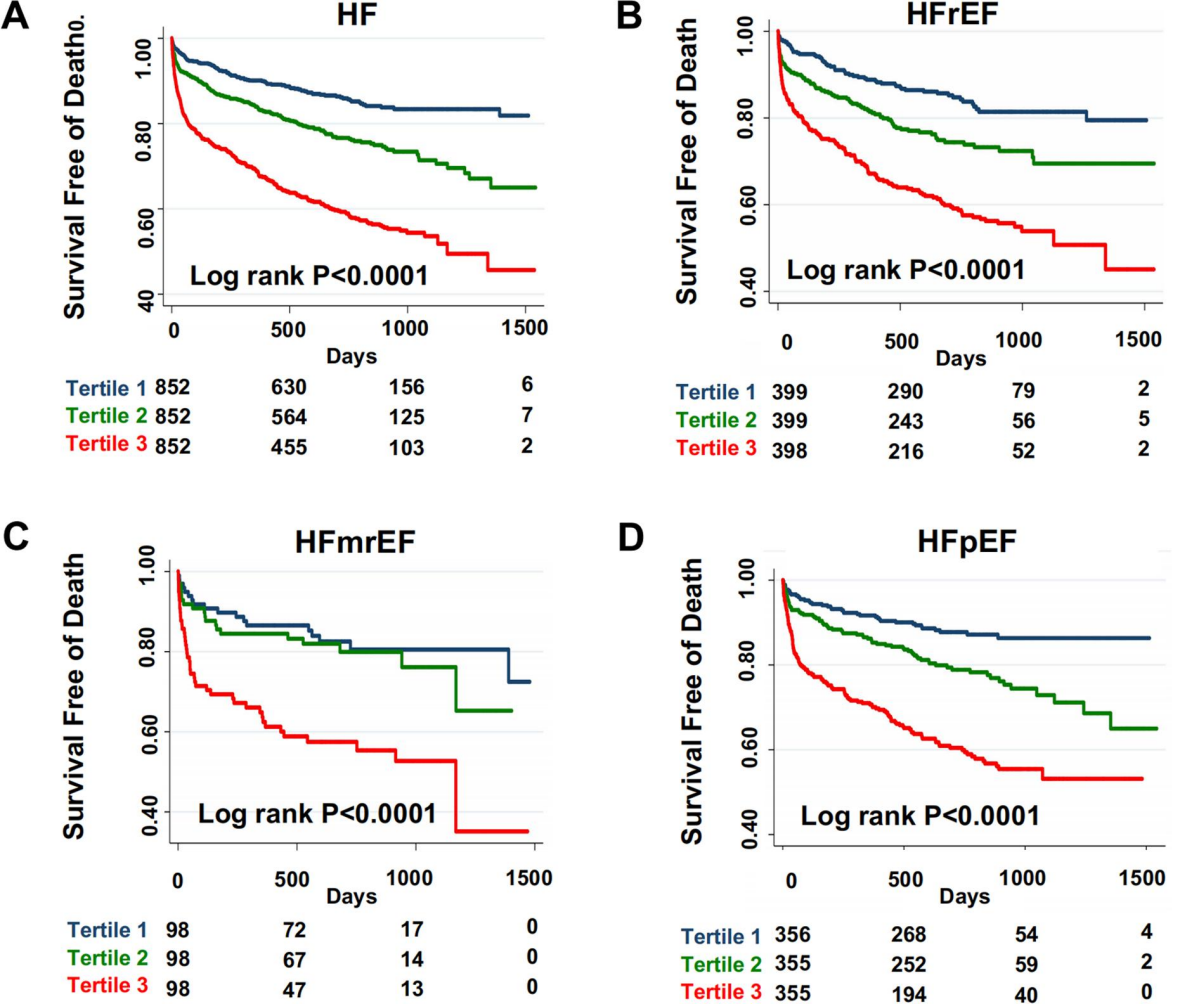
Assessment of BAR for risk stratification of mortality in patients with AHF patients. **(A)** Overall population with AHF. Kaplan–Meier survival curves illustrating the survival free of all-cause mortality stratified by tertiles of BAR. The significance of the difference among the three groups was assessed by the Log-rank test (*P* < 0.001). **(B–D)** Subgroup analysis based on ejection fraction (all log-rank *P* < 0.001). **(B)** Heart failure with reduced ejection fraction (HFrEF). **(C)** Heart failure with mildly reduced ejection fraction (HFmrEF). **(D)** Heart failure with preserved ejection fraction (HFpEF).

To evaluate the risk stratification capacity of BAR in patients with HF, we performed Kaplan–Meier survival analysis to assess event-free survival rates stratified by BAR levels. Using tertile cutoff values, patients were categorized into high- (highest tertile), intermediate-, and low-risk groups. The high-risk group demonstrated significantly worse event-free survival compared to other groups (log-rank *p* < 0.0001; Figure 1).

Notably, BAR maintained strong discriminative ability across all HF subtypes, including HFrEF, HFpEF, and HFmrEF, with significant separation of survival curves among risk strata (log-rank *p* < 0.0001 for all comparisons; Figure 1).

### Sensitivity analyses

To determine whether the prognostic significance of BAR was confounded by established cardiorenal biomarkers, we performed sensitivity analyses by excluding serum creatinine or BNP from the multivariable models. As shown in Table 6, the independent association between BAR and mortality remained robust even after excluding creatinine (HR: 1.56; 95% CI: 1.39–1.75; *p* < 0.001) or excluding BNP (HR: 1.74; 95% CI: 1.55–1.94; *p* < 0.001). These findings suggest that BAR provides unique prognostic information beyond glomerular filtration and cardiac wall stress.

**Table 6:** Sensitivity analyses for the independent association between BAR and all-cause mortality.

Adjustment model	Hazard ratio (95% CI)^a^	*p*-Value
Full adjustment model^b^	1.61 (1.43–1.72)	<0.001
Model excluding serum creatinine^c^	1.56 (1.39–1.75)	<0.001
Model excluding BNP^d^	1.74 (1.55–1.94)	<0.001

^a^ Hazard ratios (HRs) are presented per 1-unit increase in BAR.

^b^ Full adjustment model: Adjusted for age, sex, smoking, diabetes mellitus, prior myocardial infarction, prior revascularization, NT-proBNP, HDL-C, LDL-C, serum creatinine, and use of ACEI/ARB, beta-blockers, and diuretics.

^c^ Model excluding serum creatinine: Adjusted for all variables in the full model except serum creatinine.

^d^ Model excluding NT-proBNP: Adjusted for all variables in the full model except BNP.

## Discussion

In the present study, we investigated the association between the BAR level and long-term mortality in patients with AHF. Our findings demonstrated a significant correlation between BAR level and survival rate during long-time follow-up, suggesting that BAR may serve as a reliable prognostic biomarker. Given the simplicity of measuring both BUN and albumin levels, BAR offers a convenient and accessible prognostic tool.

Several studies have identified BAR as an important prognostic biomarker in various diseases, including sepsis (13), pneumonia (14), gastrointestinal bleeding (15), and lung cancer (10). For example, an observational study of 10,578 patients showed that elevated BAR (≥7.93) was associated with ICU mortality among patients with sepsis (16). Similarly, a retrospective cohort study of 1,545 patients indicated that BAR was an independent risk factor for in-hospital mortality and 90-day all-cause mortality in critically ill patients with chronic HF who were admitted to the ICU (12). A recent study also found that higher BAR was associated with an increased risk of short-term mortality or readmission in patients with chronic HF (17). However, the long-term prognostic value of BAR in AHF remained unclear. Our study demonstrates, for the first time, that after adjusting for age, sex, and previously reported risk factors of adverse outcomes, a high BAR level maintains a significant association with mortality in both the overall cohort and all three HF subtypes during a median follow-up period of 1.84 ± 0.92 years.

A central feature of AHF is systemic congestion, which is thought to contribute to multi-organ damage and increased mortality risk. It is hypothesized that elevated venous pressure in response to systemic congestion leads to renal congestion, splanchnic congestion, and liver congestion. Renal venous hypertension may increase interstitial pressure and induce a reduction in renal blood flow, potentially leading to the collapse of tubules and therefore reduced glomerular filtration rate (18). BUN is involved in renal congestion and reflects the progression of HF (19), but its predictive value for the prognosis of HF remains a subject of debate. Splanchnic congestion is proposed to disrupt the intestinal barrier and impair nutrient absorption. The resulting systemic inflammation and metabolic derangement serve as key drivers of malnutrition and low serum albumin levels in this population.

Recent evidence underscores the prognostic significance of inflammation-related and nutritional markers. For instance, the Endothelial Activation and Stress Index (EASIX)—incorporating lactate dehydrogenase, creatinine, and platelet count—has been identified as a marker of endothelial dysfunction and a contributor to early clinical deterioration in AHF (20). While EASIX highlights the role of endothelial stress, albumin and neutrophil levels remain critical markers of the complex interplay underlying systemic inflammation (21). Furthermore, the established role of inflammatory markers (22) and the demonstrated prognostic value of the C-reactive protein-to-albumin ratio (CAR) in HF further support the clinical relevance of albumin-based ratios (23). These findings align with our results, suggesting that BAR serves as an integrative surrogate for systemic inflammatory and metabolic derangements driving poor outcomes in patients with AHF (Reviewer 2, No.10).

Albumin is secreted by hepatocytes, and its concentration reflects the balance of synthesis, consumption, distribution, and external losses (24). In patients with AHF, severe liver congestion could cause redistribution of albumin from the vascular to interstitial space, and even low production of albumin. In addition, hypoalbuminemia is an independent risk factor for poor HF prognosis. Therefore, BAR likely serves as a surrogate integrative marker, capturing the intersection of hemodynamic congestion, renal dysfunction, and metabolic impairment in patients with AHF.

A notable finding of our study is the consistent prognostic performance of BAR across the LVEF spectrum (HFrEF, HFmrEF, and HFpEF). While these phenotypes differ in their primary myocardial mechanics, they share a common terminal pathway characterized by hemodynamic congestion and neurohormonal activation (25). Elevated BAR likely reflects impairment of the “cardio-renal-hepatic” axis, particularly, renal venous hypertension and splanchnic congestion, which are prevalent drivers of mortality regardless of ejection fraction.

In the HFpEF population, where systemic comorbidities often drive prognosis more than hemodynamic impairment alone, BAR may be informative as it integrates renal engagement (via BUN) with systemic inflammation and nutritional status (via albumin). However, it is important to acknowledge that serum albumin is sensitive to non-cardiac factors such as chronic liver disease, subclinical inflammation, and malnutrition. As highlighted in recent studies, these non-cardiac drivers are prevalent in HFpEF and can confound the interpretation of albumin-based biomarkers (26). In patients with HFpEF, these factors may confound the association between BAR and mortality. While we excluded patients with active malignancy, other underlying conditions might have contributed to the observed risk. Thus, BAR should be interpreted as a global marker of clinical vulnerability and multiorgan impairment rather than a heart-specific indicator, particularly in the context of HFpEF.

In evaluating these associations, alternative explanations such as residual confounding and reverse causation must be considered. Although we adjusted for major clinical variables, unmeasured factors such as baseline frailty, subclinical hepatic cirrhosis, or undisclosed malignancy could contribute to both elevated BAR and increased mortality, representing residual confounding. Furthermore, reverse causation cannot be entirely ruled out in this retrospective cohort; an elevated BAR might simply be a downstream manifestation of a rapidly deteriorating physiological state (a “preterminal” marker) rather than a causal driver of long-term cardiovascular decline. These possibilities underscore the need for prospective studies with serial measurements to clarify the temporal and causal nature of the BAR–mortality link.

High BAR results from either increased BUN or decreased albumin levels, with both BUN and serum albumin being well-established biomarkers associated with HF. In patients with AHF, fluid retention and activation of the sympathetic nervous system and renin–angiotensin–aldosterone system induce renal dysfunction, which aggravates the clinical outcome. BUN is a common indicator reflecting renal function and is associated with higher mortality in patients with HF (27). For example, in the PROTECT trial, elevated BUN level alone was significantly associated with 180-day mortality in patients with AHF (28). Moreover, during hospitalization, persistent high BUN levels are associated with an increased risk of cardiovascular death and HF readmission (29). Cleland et al. reported that BUN is a better prognostic marker of HF and reflects the severity of renal dysfunction in cachexia (28). However, other studies have found no predictive value of BUN for short- or long-term mortality in patients with HF (30, 31), highlighting its controversial role as a standalone prognostic biomarker in AHF.

Hypoalbuminemia is commonly seen in patients with HF, and several studies have demonstrated that it can be an independent risk factor for poor prognosis. Data from a retrospective cohort study showed that, among 1,365,529 adults hospitalized for congestive HF, 88% had a secondary diagnosis of hypoalbuminemia (32). Moreover, patients who were admitted with HF and concomitant hypoalbuminemia had approximately twice the risk of in-hospital mortality compared with patients with normal albumin levels. Furthermore, in advanced systolic HF, hypoalbuminemia has been found to be independently associated with increased all-cause mortality during 5-year follow up (13). Prenner et al. (33) found the lower albumin is not only a powerful risk predictor of adverse outcomes in patients with HFpEF, even after adjustment for several risk scores, but also an integrated marker of various adverse processes in HFpEF, including inflammation, arterial stiffness, and subclinical liver dysfunction.

In conclusion, higher BAR levels, driven by the interplay between renal dysfunction and impaired nutritional status, are associated with poor long-term outcomes in AHF patients, offering superior risk stratification compared to its individual markers. This study provides novel evidence to support the prognostic value of BAR in terms of long-term mortality in patients with AHF. BAR can be calculated from BUN and albumin levels, which are routinely collected biomarkers in patients with AHF. Its simplicity and strong prognostic value position BAR as a very useful marker in patients with AHF.

### Limitation

Several limitations of our study should be acknowledged. First, as a single-center study conducted in China, our findings are preliminary and may not be generalizable to non-Asian populations due to differences in healthcare systems and nutritional status. External validation in broader, diverse cohorts is essential before clinical adoption. The use of BAR tertiles is primarily exploratory; as this single-center study aimed to demonstrate a risk gradient rather than define clinical thresholds, these tertiles are not intended for immediate bedside decision-making. Second, while we adjusted for key confounders, other unmeasured variables (e.g., diet, compliance) could influence BAR level. Third, the ratio's components (e.g., albumin) are non-cardiac-specific; concurrent conditions (e.g., liver disease) may confound interpretation. Fourth, BAR was measured only at admission, which may not capture its dynamic changes during hospitalization. This single-point assessment introduces potential regression dilution bias, which might underestimate the true association between BAR and long-term mortality. Fifth, while we hypothesized mechanistic links between BAR and congestion or inflammation, these remain hypothesis-generating as direct hemodynamic or cytokine measurements were not available. Finally, the lack of cause-of-death adjudication led us to use all-cause mortality as the primary endpoint. Although this avoids competing risk bias in this elderly, multimorbid cohort, it limits our ability to isolate cardiovascular-specific effects.

### Summary

BAR is a readily calculable, potentially impactful biomarker that is independently associated with long-term mortality across HF phenotypes. Its integration into existing risk scores could enhance prognostic precision and personalize management.

## Data Availability

The original contributions presented in the study are included in the article/Supplementary Material, further inquiries can be directed to the corresponding authors.
